# Orthopaedic surgeons can play important role in identifying victims of domestic violence in the emergency department – narrative review of Brazilian literature

**DOI:** 10.1097/MD.0000000000031461

**Published:** 2022-12-16

**Authors:** Vincenzo Giordano, Carolina Giordano, Isadora Maria Lopes, Robinson Esteves Pires, Alexandre Godoy-Santos, Peter V. Giannoudis

**Affiliations:** a Serviço de Ortopedia e Traumatologia Prof. Nova Monteiro, Hospital Municipal Miguel Couto, Rio de Janeiro, Brazil; b Clínica São Vicente, Rede D’or São Luiz, Rio de Janeiro, Brazil; c Clínica da Família Maria do Socorro Silva e Souza, Secretaria Municipal de Saúde do Rio de Janeiro, Clínica da Família Estácio de Sá, Secretaria Municipal de Saúde do Rio de Janeiro, Rio de Janeiro, Brazil; d Departamento de Ortopedia, Universidade Federal de Minas Gerais (UFMG), Belo Horizonte, Brazil; e Hospital das Clínicas HCFMUSP, Faculdade de Medicina, Universidade de São Paulo, São Paulo, Brazil; f Hospital Israelita Albert Einstein, São Paulo, Brazil; g Academic Department of Trauma & Orthopaedic Surgery, School of Medicine, University of Leeds, Leeds, United Kingdom.

**Keywords:** battered women, domestic violence, emergency, intimate partner violence, orthopedics, violence against women

## Abstract

Over the last year, with the social isolation imposed by the coronavirus disease pandemic, there has been a significant increase in complaints associated with physical violence against women. In the present study, an exploratory literature review was carried out on the role of the on-call orthopedic surgeon when faced with a suspicion of domestic violence, in accordance with Brazilian legislation. The main objective of the study was to show the role of this specialist in identifying victims of domestic violence by recognizing their profiles and associated risk factors. The secondary objectives were to demonstrate the most common skeletal and non-skeletal injuries in this type of violence and to present a quick and practical guide on how to identify, approach, and manage cases of domestic violence against women. The findings revealed that the main aggressors were close partners, such as spouses and ex-spouses. Young adult women, black or multiracial, and low socioeconomic status are major risk factors for intimate partner violence. Head and neck injuries are the most frequently observed lesions in this population, with more than one-third of victims reporting falls. Musculoskeletal injuries are present in up to 42% of victims of domestic violence, occurring predominantly in the upper limbs and chest, and are the leading cause of death in women aged 1 to 34 years. A practical guide for orthopedic surgeons who work in emergency departments is proposed, with basic information about their role and responsibility in identifying potential victims of intimate partner violence.

## 1. Introduction

Domestic violence against women has become a relevant and prevalent public health problem and is increasingly included in the agenda of discussions in Brazilian society. Guimarães and Pedroza pointed out that sexist and patriarchal cultural values, historically structured in the Brazilian society, are associated with the serious recurrence of violence committed against women and the serious inequalities of power and rights faced by them.^[1]^ Despite the creation of laws, highlighting Law No. 11.340 of August 7, 2006 (Law Maria da Penha),^[2]^ and preventative actions, domestic violence against women continues to occur deliberately throughout the country, regardless of ethnicity, gender, color, age, religion, social status, or educational level.

According to the World Health Organization, domestic violence against women results from the “behavior of a partner or ex-partner that causes physical, sexual, or psychological harm, including physical aggression, sexual coercion, psychological abuse, and control behaviors.” Global estimates indicate that approximately one in three women have experienced physical and/or sexual violence by their partner or third parties during their lifetime, and these are the main victims of homicides committed by intimate partners.^[3]^ In addition, women who have suffered or suffer violence are more likely to develop depression, anxiety, insomnia, and eating disorders, and are more prone to alcohol and illicit drug consumption and suicide attempts. The long-term effects on physical health observed in these women include headache, gastrointestinal disorders, and fibromyalgia.^[3]^

Noteworthy, orthopedic injuries are the leading cause of death in females between one and 34 years old worldwide.^[4,5]^ Della Rocca et al^[5]^ revealed that one in 50 women presenting to an orthopedic clinic with an injury have been injured from intimate partner violence (IPV), with musculoskeletal lesions being the second-most prevalent injury type after head and neck injuries. Additionally, multiple injuries have been strongly associated with IPV, including sprains, fractures/dislocations, lacerations, stab wounds, and chest contusions, which are normally more severe than blunt force head, facial, and neck injuries.^[6]^ Of interest, it has been found that lacerations and fractures are significantly associated with IPV.^[7]^

Despite the high number of cases of IPV, there is a lack of knowledge on the part of orthopedic surgeons in identifying these patients, which may stem from lack of education and personal uneasiness.^[8]^ The problem is made more critical by the lack of notification by female victims of domestic violence.^[9]^ Approximately one-third of injured women presenting to fracture clinics have experienced some form of intimate partner violence in the past year.^[3,8]^ It has been shown that multiple psychosocial factors, such as fear, stigma, threat, shame, and guilt, and negative experiences in the past, such as discrediting, prevent the victim of domestic violence from reporting what happened to the authorities or to the health team when they present themselves to an emergency department (ED).^[10]^ In this context, the type of ED care can define different outcomes in their history, since health services are considered the gateway for women to the support system.

In the present study, an exploratory literature review was carried out on the role of the on-call orthopedic surgeon when faced with a suspicion of domestic violence, in accordance with Brazilian legislation. The main objective of the study was to show the importance and role of this specialist in identifying victims of domestic violence by recognizing their profiles and associated risk factors. The secondary objectives were to describe the most common skeletal and non-skeletal injuries in this type of violence and to present a quick and practical guide on how to identify, approach, and manage cases of domestic violence against women, thus reducing the ongoing damage caused by the aggressions suffered by their intimate partners and potentially preventing the death of many women.

## 2. Material and methods

### 2.1. Research method and design

We conducted a narrative literature review on the role of orthopedic surgeons in the ED regarding the identification of potential victims of domestic violence against women and their treatment, including notification to legal authorities to act in these scenarios. The Scale for the Assessment of Narrative Review Articles was followed during the search and reporting phases. For the purposes of this study, we used PubMed/MEDLINE and Google Scholar databases from January 2006 to January 2021. The initial criteria for inclusion in the research are as follows: scientific articles written in English and/or Portuguese, published in the last 15 years, with information on musculoskeletal injuries, epidemiology, and Brazilian legislation on victims of domestic violence. In PubMed/MEDLINE, the terms used were “intimate partner violence AND fractures,” “intimate partner violence AND orthopedic surgeon,” “intimate partner violence AND emergency room,” and “intimate partner violence AND trauma.” In Google Scholar, we searched for Brazilian articles dealing with IPV published in medical journals not indexed in PubMed/MEDLINE; however, we present relevant data for this narrative review. The terms used were “violence against women injuries” and “domestic violence” all written in Portuguese for the purpose of the research. Some studies dealing with IPV have been included to contextualize the problem and show the importance of the subject. However, these studies were excluded from analysis of the results and discussion. Studies that did not address musculoskeletal injuries or the role of the on-call orthopedic surgeon on IPV were excluded. In addition, we excluded case series, case reports, and expert opinions.

### 2.2. Data collection and analysis

All studies selected were read from the paper abstracts, with full texts included when needed to identify the relevance of the paper to our research. All duplicates were removed. Two investigators (CG and IL) independently and in duplicate reviewed the selected full-text articles for inclusion. After consensus, the reference lists of the included articles were reviewed for additional studies and subsequently appraised by the same two investigators for inclusion. Bibliographic data in sampled publications were analyzed to identify the following outcome: victim profile and risk factors, aggressors’ profile, orthopedic approach, and screening protocol for IPV victims in the ED.

## 3. Results

Overall, of the 4749 papers screened in PubMed/MEDLINE, seven papers met the inclusion criteria and formed the basis of this review.^[11–17]^ Of the 230,800 results obtained in Google Scholars, six articles met the inclusion criteria and formed the basis of this review.^[18–23]^

### 3.1. Victims’ profiles and risk factors

The data showed a higher prevalence of domestic violence in young adults, between 18 and 40 years of age, with an incidence peak around 30 years, although a considerable number of cases have been observed during adolescence (between 12 and 19 years of age).^[3,11–13,18]^ The prevalence of IPV once in a lifetime was 34.5%.^[13]^ It has been shown childhood sexual abuse as an independent risk factor for intimate partner sexual violence in adult life.^[14]^ There was a multiracial prevalence, with heterogeneous distribution among white, black, and brown women depending on the state of the country and if the victim lived in a rural or urban area.^[11,13,14]^ Most declared to be Catholic, married or cohabiting with a partner.^[13,14]^ Most women indicated their husband or partner as the head of the family, lived in their own house, and presented socioeconomic status C or D—monthly income from one to three minimum wages per month.^[13,14]^ The chance of experiencing IPV increased by almost four times for separated or divorced women.^[13]^ There was a prevalence of violence against single women, especially those who were single mothers.^[3,11–14,18]^ Few women had completed higher education (more than 12 years of study in Brazil) and most of them had no paid work.^[3,11–14,18]^ The lower the level of education, the greater the chance of violence.^[13]^

Personal and environmental factors associated with victims showed a direct relationship with cases of domestic violence, such as exposure to child abuse, experience of family violence, mental illness, harmful use of alcohol and illicit drugs, attitudes of acceptance of violence, difficulty of communication between partners, loss of financial resources, and the first year after the end of the relationship/divorce.^[11–14,18]^ Almost half of the episodes of intimate partner violence occurred during weekends and almost 40% during the nighttime.^[11]^ In the last year, the social isolation caused by the coronavirus disease pandemic also entered this group of risk factors for domestic violence.^[15,19]^

### 3.2. Aggressors’ profile

Most cases of IPV were practiced by a single man.^[20,21]^ Most were young adults, married, with low education and paid work.^[21]^ However, there were situations in which the violence was perpetrated by another woman and/or there was more than one aggressor.^[12]^ Up to 50% of the cases were inflicted by intimate partners, mainly spouses or ex-spouses, and more than 90% of the occurrences were practiced by people close to the victim, such as neighbors, and siblings or other family members.^[3,13,14,20,21]^ Physical violence was perpetrated by the majority of the arrested aggressors, being practiced alone or associated with other types of violence, mainly psychological.^[21]^ Most aggressors were undertaking paid work, mainly informal jobs.^[18,21,22]^ Alcohol consumption was observed in up to 48% of aggressors.^[11]^ A third had files or records of other police arrests, with up to 60% of these resulting from domestic violence.^[21]^ In one study, it was found that 89.3% were released on warranty.^[21]^

### 3.3. Orthopedic approach and screening protocol for IPV victims in the ED

Musculoskeletal injuries mainly in the upper limbs and trunk are the second most prevalent type of injury after head and neck injuries.^[14,15,21]^ The most frequent mechanism of IPV was blunt trauma, accounting for up to 70% of the injuries.^[11,14,15,21]^ As a result, bruises were the main finding in most women.^[11,18]^ Fractures were observed in 9.7%, sprains in 15.6%, and lacerations in 16.9% of cases. Non-skeletal injuries to the internal organs were observed in 14.4% of the patients. Sexual violence against women was reported in 12.9% of the cases, mainly in women 20 years or older.^[20]^ In the upper limbs, the fingers and forearm bones (particularly the ulna) were common sites of IPV injury. In the trunk, rib fractures and posterior arch vertebral fractures were observed. Finally, in the lower limbs, the most affected regions were the feet and ankles; however, musculoskeletal injuries in this body segment occurred less frequently. It was observed that most IPV injuries did not occur in isolation, generally being accompanied by other injuries in different parts of the body or in the same region.^[18]^ Mattos et al^[18]^ observed that head and limbs injuries occurred either alone or associated with chest and neck injuries, however, they did not present the percentage of the associated injuries. Garcia and Silva found 14.8% polytrauma patients among 350 female victims, however this percentage was presented together with cranial injury.^[11]^ No other author reported on polytrauma patients in their series, nor the Injury Severity Scale was calculated in these studies^[12–16,18–23]^

No study has referred to or presented a screening protocol for IPV injuries in patients with skeletal trauma in the ED.^[11–16,18–23]^ some authors mentioned the need to develop and implement more effective, intersectoral, and interdisciplinary actions to combat and prevent IPVs in Brazil; however, no specific protocol was found.^[11,21]^ Of relevance, approximately a quarter of victims had sought care at another medical institution for the same occurrence before their injuries were considered because of IPV.^[11]^

## 4. Discussion

Although IPV can affect anyone, regardless of gender, religion, ethnicity, or socioeconomic status, studies allow for a well-defined profile of victims in Brazil: young adult women, on average around 30 years of age, multiracial, with low income, and low educational level.^[3,11–23]^ This can be explained by the greater social vulnerability associated with these groups, both due to the historical debt of the patriarchal society and the greater difficulty in accessing information. Of interest, Garcia and Silva^[11]^ and d’Oliveira et al^[14]^ found that white women generally had a higher level of education and financial autonomy than black and brown victims. North American and European authors have revealed a higher prevalence among black and brown women, with a small proportion of white victims.^[24,25]^ In Brazil, single mothers appear as great victims, although separated or divorced women were shown to present a high risk of IPV as well. A considerable number of cases have been observed between 12 and 19 years of age, highlighting the deleterious effects of maltreatment on children and adolescents, with permanent and often irreversible consequences on women’s health. Previous experience of family violence, mental illness, harmful use of alcohol and illicit drugs, attitudes toward acceptance of violence, difficulty in communication between partners, loss of financial resources, and the first year after termination of the relationship/divorce are known risk factors for IPV. It is noteworthy that, regardless of whether the data collected point to a specific victim profile, this does not exclude patients who do not fit these patterns, mainly because most studies considered the female sex, not considering the gender identity, excluding women transsexuals, which can either decrease the accuracy of the studies or mask the real number of IPV. Interestingly, using the National Intimate Partner and Sexual Violence Survey, Sugg found a higher prevalence of physical violence, rape, and lifelong persecution among bisexual women compared to lesbians or heterosexuals, which may suggest this sexual orientation as a risk factor.^[10]^ Finally, it is worth remembering that the social isolation imposed by the coronavirus disease pandemic increased IPV cases all over the world, including Brazil.^[15,19,26–28]^

Few studies have assessed the profile of the aggressors, which can be explained either by the focus on the victim or the patients not expressing the desire or being afraid to expose those who attacked them.^[12,16,18,20,21,23–25,29]^ Della Rocca et al^[5]^ reported that lack of reliable housing, inability to obtain counseling, financial resources and child custody, and lack of knowledge about legal issues may also be associated with victims’ vulnerability. The Brazilian reported that lack of reliable housing, inability to obtain counseling, financial resources and child custody, and lack of knowledge about legal issues. Vieira et al^[13]^ found that the risk of violence in women increased 59% in cases where the partner used alcohol frequently and almost six times when the aggressor used other drugs. The aggression occurs in most cases by a single man, usually close to the victim, which includes spouses, ex-spouses, family members, or neighbors. For this reason, it is essential to understand the patient’s social core. However, there are situations, albeit to a lesser extent, in which violence can occur by more than one partner or even practiced by another woman. Of interest, many authors have shown severe human rights violations, with an increased prevalence of IPV, sexual slavery, depression, and posttraumatic stress disorder syndrome in war-affected populations.^[30–35]^ This can be explained at least in part by cultural factors, economic disparities, and religious characteristics. It has been stated that religious values heavily influence policymaking, potentially affect many social aspects, and have direct implications on many social and demographic phenomena.^[36,37]^ Brazil is considered a multi-religious country, with Catholics, Pentecostals, and neo-Pentecostals being the largest religious affiliation.^[38]^ Steiner et al^[39]^ investigated a cohort of women attended in a Brazilian public hospital in Brazil between 2018 and 2019 and found that Pentecostal religion was more prevalent in women who suffered violence than those who did not. The association of religious affiliation and alcohol-mediated violence was reported by Gonçalves et al.^[37]^

Up to 50% of the injuries occur through blunt instruments, such as truncheons, pieces of wood, baseball bats, and many times a punch.^[10,11,17,18,24,25,29,40]^ It was demonstrated that victims of IPV requiring hospital admission spent an average of five days in the hospital, with more than 25% requiring admission to the intensive care unit.^[29]^ Unfortunately, the majority of victims of IPV who present in an ED with blunt injuries attribute them to events not related to aggression, such as falls, for example.^[40,41]^ The greatest number of IPV injuries occur to the head, especially the facial bones and teeth, and the neck, reflecting the symbolic character of humiliation that the aggressor imprints on the woman’s face, making the lesion visible and thereby changing the characteristics that infer female beauty, an attribute that is highly valued socially.^[17,42,43]^ In general, injuries to these sites raise greater suspicion or are easier to diagnose, as they are strongly associated with physical aggression mechanisms.^[10,18]^ Importantly, most of these injuries are not unique, usually being accompanied by other injuries, which makes essential to actively search for injuries in other areas of the body, which may not have been reported by the victim, such as certain orthopedic injuries that have been related to abuse. Musculoskeletal injuries in the upper limbs and trunk were the second-most prevalent injury type reported in Brazilian studies.^[14,15,21]^ Assessing electronic medical records of 185 patients referred to the IPV support program from the ED of Brigham and Women’s Hospital in Boston, George et al^[41]^ reported that acute fractures involving the face or skull (range, *P* < .01, *P* = .05), and chronic fractures affecting the extremities and nasal bone (*P* < .01 and *P* = .05, respectively) were more frequent in the IPV group than in the control group. In their study, victims of IPV were more likely to sustain upper-extremity fractures than lower-extremity fractures, which was also observed in Brazilian studies. Garbin et al^[17]^ state that trauma to the upper limbs, especially to the fingers and forearm (particularly the ulna), and to the ribs, should always be suspected as arising from IPV, and may represent a defensive behavior on the part of the victims, who tried to protect the face of blows, or that they were stepped on or kicked after they were defenseless by the initial aggressions. Other authors observed a high number of abrasions, lacerations, and sprains, including neck and back sprains.^[4,24]^

In this scenario, the role of the orthopedic surgeon who works on the front lines of emergency care is fundamental, questioning in a non-intimidating and judgment-free manner in which women suspected of IPV, taking care not to neglect or misinterpret these patients.^[41,44,45]^ The earlier the detection of abuse, the more preventable serious injuries and consequences can be, including mutilation and death.^[46]^ PRAISE Investigators reported that more than two-thirds of women support that all women who present after trauma in an orthopedic emergency should be asked about IPV.^[40]^ Sprague et al^[9]^ observed that 74% of their respondents agreed that orthopedic surgeons should look for the cause of the patient’s injury and 61% agreed that these professionals can help victims of IPV. Therefore, a high level of suspicion is necessary to allow orthopedic surgeons to perform complementary examinations or imaging interventions that enable the detection of victims of IPV. Milner et al^[46]^ observed that there are some notable “red flags” that are considered particularly sensitive signs of abuse, such as explicit denial of trauma in conflict with clinical symptoms, particularly when given by the patient, and explanations that are inconsistent with the observed trauma. Since 1992, the Joint Commission on the Accreditation of Healthcare Organizations has mandated that all hospitals in the US have policies and procedures for the early identification and referral of victims of abuse.^[47]^ Several screening protocols and tools have been proposed to assist orthopedic surgeons in the ED when IPV is suspected.^[5,9,10,29,40]^ Overall, these studies suggest the need for personal interviews, with written questions asked by experts and answered in a reserved and safe environment, away from the possible aggressor. In addition, these studies point out the importance of the interviewer being of the same sex and speaking the same language as the victim, never using stigmatizing words or terms, and expressing her concern about the impact of violence on the victim’s health. A hazard assessment tool was developed by Campbell et al^[48]^ to see the probability of future serious injury or death in victims of IPV, with a sensitivity of 83%. Sugg suggests that if the patient is classified as “at risk of serious injury” using this assessment tool, the physician should fully discuss the situation with the victim and encourage her to make a strong safety plan.^[10]^ however it has been seen that the majority of hospitals in the US are still not compliant with this recommendation.

In Brazil, to the best of our knowledge, no single IPV screening tool has been established for the assessment of intimate partner violence, making it critical to adapt or create a tool that meets the cultural and socioeconomic characteristics of the Brazilian population. Therefore, the identification of IPV by healthcare practitioners remains very low in this country, which results in continued abuse, with a possible fatal outcome. Despite the lack of an existing screening tool, Brazilian current legislation brings the concept of compulsory notification in cases of suspected violence against women, with all health professionals overseeing this function.^[2]^ In addition, article 25 of the Code of Medical Ethics, published by the Brazilian Federal Council of Medicine through Resolution 1931/2009, prohibits physicians from denouncing torture or degrading and inhumane procedures, them, as well as colluding with those who carry them out, or providing means, instruments, or knowledge to facilitate them.^[49]^ In this sense, the article establishes the physician’s responsibility to report cases of violence, through notification aimed at protecting the patient. Unfortunately, this is slightly different in practice. Hasse and Vieira^[50]^ observed a great lack of preparation on the part of health professionals, especially physicians, when conducting suspected cases of violence against women. The professionals’ difficulty in recognizing violence as a possible cause for several injuries that they attend daily seems to be associated with the epidemiological lack of knowledge about the violence itself, which automatically generates enormous difficulty in reflecting or even blocking the issue. Some hypotheses can be raised to justify this difficulty in recognizing possible victims of IPV, such as the lack of interest in professionals in understanding violence as a public health issue, the lack of study on the subject during curriculum formation, and fear of reprisals for part of the aggressor. Almeida et al^[51]^ corroborate these ideas when they point out that most health disciplines do not include training in aspects related to domestic violence in their curricula or permanent education programs, so that professionals are not prepared to provide effective health care for the victim. These authors suggest that the lack of institutional protocols that guide professional performance and knowledge about the service network can generate insecurity in the handling of IPV cases by health professionals. The lack of technical knowledge on the part of health professionals about IPV was also observed by Della Rocca et al.^[5]^ These authors suggest using the “EDUCATE” training program (http://www.ipveducate.com/home), which includes online video lessons, bimonthly updates, and IPV awareness posters. In Brazil, Almeida et al^[51]^ proposed the adoption of technology as an ally of medical education focused on IPV through the computer games “Serious Games,” which contributes to the association between theory and practice, consequently leading to a change in the care provided to victims of IPV by health care professionals.

As in many parts of the world, in Brazil, orthopedic surgeons play an active role in ED care, as frontline health professionals with critical roles in improving patient safety. In this context, it is essential that these professionals understand the severity of IPV against women and suspect of any skeletal injury in the presence of “red flags” indicative of physical abuse.^[46]^ An evidence-based approach should be followed in order to allow orthopedic surgeons to effectively identify and help patients who have experienced IPV, including case-finding protocols and support plans, in particular considering the characteristics of victims and policies aimed at combating alcohol and illicit drugs. Therefore, in addition to reporting and treating fractures and other skeletal injuries, it is also possible to act to change the aggressors’ violent behavior and promote gender equality, especially regarding women’s economic, emotional, and social emancipation. Thus, the authors propose the adoption of a screening protocol for suspected IPV cases in the clinical setting of fractures based on Brazilian and international data (Table 1). The first part (**identifying potential victims**) brings demographic, ethnic, and socioeconomic characteristics of patients at risk, the most common data on the aggressors, the most frequent complaint on hospital admission, and clinical and imaging findings that may suggest IPV. In the second part (**approaching the victim**), a rational and effective way of collecting the history of the potential victim of IPV is suggested, including where the anamnesis should be carried out, the type of questions that should be asked, and the attitude of the interviewer (doctor or nurse). Finally, the third part (**conducting the case**) shows options for communication channels and protection of IPV, and necessary documents that must be filled in by the healthcare professional who assists the patient. Milner et al^[46]^ stated that by increasing availability and access to resources related to intimate partner violence and other types of violence against individuals considered vulnerable, more people can recognize and report suspected abuse.

**Table 1:** Proposed screening protocol for identifying and managing suspected IPV cases sustaining skeletal and non-skeletal injuries at the ED.

Parts of the screening tool	Findings, acute actions, and management
Identifying potential victims	1. Young adult women 2. White skin color, higher level of education, and financial autonomy 3. Black and brown skin color, low level of educational, and low income 4. Unstable family core 5. Partner with history of alcohol and illicit drug abuse 6. Explicit denial of trauma in conflict with clinical symptoms and explanations that are inconsistent with the observed trauma 7. Blunt injuries to the head (face and teeth) and the neck 8. Fractures (upper limbs and trunk), hematomas, bruises, and sprains 9. Imaging exams showing multiple associated skeletal and non-skeletal injuries
Approaching the victim	1. Prefer interviewers of the same sex, who speaks the same language 2. Take the victim to a private, safe, and comfortable environment 3. Never use stigmatizing words or terms 4. Discuss about IPV in a direct but welcoming way 5. Avoid value judgment 6. Avoid blaming the victim and validate their suffering
Conducting the case	1. After confirmation of the occurrence of IPV, the victim must be instructed to go to the police station to register the occurrence 2. When there is a suspicion of IPV, regardless of whether it is confirmed or not, the physician must fill in the SINAN notification form 3. Guide the victim about the existence of the Women’s Assistance Center, calling 180 4. Guide the victim about the existence of the Law Maria da Penha (no. 11.340 of August 7, 2006)

Source: Authors, 2021.

ED = emergency department, IPV = intimate partner violence.

## 5. Conclusion

Recent studies point to the growing high incidence of domestic violence against women in Brazil, which seems directly related to the existence of a patriarchal organization in social relations between the sexes, with women being constantly exposed to aggression. Orthopedic surgeons play a critical role in the identification and management of IPV victims, and should go beyond the scope of the usual treatment for skeletal injury. Musculoskeletal injuries are present in up to 42% of victims of domestic violence. The main aggressors are close partners, such as spouses and ex-spouses. Young adult women, black or multiracial, and low socioeconomic status are major risk factors for IPV. The proposed screening protocol for identifying and managing suspected IPV cases sustaining skeletal injuries at the ED potentially allows orthopedic surgeons along with other health professionals to identify and protect victims of IPV from their aggressors, thus preventing violence and its consequences.

## Author contributions

**Conceptualization:** Vincenzo Giordano, Carolina Giordano, Isadora Maria Gonçalves Lopes.

**Data curation:** Carolina Giordano, Isadora Maria Gonçalves Lopes.

**Formal analysis:** Vincenzo Giordano, Carolina Giordano, Isadora Maria Gonçalves Lopes.

**Investigation:** Vincenzo Giordano, Carolina Giordano, Isadora Maria Lopes.

**Methodology:** Vincenzo Giordano.

**Project administration:** Vincenzo Giordano, Carolina Giordano, Isadora Maria Lopes.

**Resources:** Vincenzo Giordano, Carolina Giordano, Isadora Maria Lopes.

**Supervision:** Vincenzo Giordano, Peter V. Giannoudis.

**Validation:** Vincenzo Giordano, Carolina Giordano, Isadora Maria Lopes, Robinson E. Pires, Alexandre Godoy-Santos, Peter V. Giannoudis.

**Visualization:** Vincenzo Giordano, Carolina Giordano, Isadora Maria Lopes, Robinson E. Pires, Alexandre Godoy-Santos, Peter V. Giannoudis.

**Writing – original draft:** Vincenzo Giordano.

**Writing – review & editing:** Carolina Giordano, Isadora Maria Lopes, Robinson E. Pires, Alexandre Godoy-Santos, Peter V. Giannoudis, Vincenzo Giordano.
